# What is the effect of perioperative intravenous iron therapy in patients undergoing non-elective surgery? A systematic review with meta-analysis and trial sequential analysis

**DOI:** 10.1186/s13741-018-0109-4

**Published:** 2018-12-12

**Authors:** Akshay Shah, Antony J. R. Palmer, Sheila A. Fisher, Shah M. Rahman, Susan Brunskill, Carolyn Doree, Jack Reid, Anita Sugavanam, Simon J. Stanworth

**Affiliations:** 1 Radcliffe Department of Medicine, John Radcliffe Hospital, University of Oxford, Level 4 Academic Block, Oxford, OX3 9DU UK; 2 Nuffield Department of Orthopaedics, Rheumatology, and Musculoskeletal Sciences, University of Oxford, Oxford, UK; 3 Frimley Health NHS Foundation Trust, Camberley, Surrey GU16 7UJ UK; 4 Systematic Review Initiative, NHS Blood & Transplant, Oxford, UK; 5 Brighton and Sussex University Hospitals NHS Trust, Brighton, UK

**Keywords:** Anaemia, Iron, Surgery

## Abstract

**Background:**

Guidelines to treat anaemia with intravenous (IV) iron have focused on elective surgical patients with little attention paid to those undergoing non-elective/emergency surgery. Whilst these patients may experience poor outcomes because of their presenting illness, observational data suggests that untreated anaemia may also be a contributing factor to poor outcomes. We conducted a systematic review to investigate the safety and efficacy of IV iron in patients undergoing non-elective surgery.

**Methods:**

We followed a pre-defined review protocol and included randomised controlled trials (RCTs) in patients undergoing non-elective surgery who received IV iron. Primary outcomes were all-cause infection and mean difference in haemoglobin (Hb) at follow-up. Secondary outcomes included transfusion requirements, hospital length of stay (LOS), health-related quality of life (HRQoL), mortality and adverse events.

**Results:**

Three RCTs (605 participants) were included in this systematic review of which two, in both hip fracture (HF) patients, provided data for meta-analysis. Both of these RCTs were at low risk of bias. We found no evidence of a difference in the risk of infection (RR 0.99, 95% CI 0.55 to 1.80, *I*^2^ = 9%) or in the Hb concentration at ‘short-term’ (≤ 7 days) follow-up (mean difference − 0.32 g/L, 95% CI − 3.28 to 2.64, *I*^2^ = 37%). IV iron did not reduce the risk of requiring a blood transfusion (RR 0.90, 95% CI 0.73 to 1.11, *p* = 0.46, *I*^2^ = 0%), and we observed no difference in mortality, LOS or adverse events. One RCT reported on HRQoL and found no difference between treatment arms.

**Conclusion:**

We found no conclusive evidence of an effect of IV iron on clinically important outcomes in patients undergoing non-elective surgery. Further adequately powered trials to evaluate its benefit in emergency surgical specialties with a high burden of anaemia are warranted.

**Trial registration:**

This systematic review was registered on PROSPERO (CRD42018096288)

**Electronic supplementary material:**

The online version of this article (10.1186/s13741-018-0109-4) contains supplementary material, which is available to authorized users.

## Background

Perioperative anaemia is common in surgical patients and associated with adverse outcomes (Musallam et al. 2011; Baron et al. 2014). The principles of patient blood management (PBM), which emphasise early diagnosis and treatment of anaemia, have been predominantly applied to patients undergoing *elective* surgery (‘Practice Guidelines for Perioperative Blood Management An Updated Report by the American Society of Anesthesiologists Task Force on Perioperative Blood Management*’ 2015; Kotze et al. 2015; Munoz et al. 2018) with little attention paid to patients undergoing *non-elective* or emergency surgery. Yet, patients undergoing non-elective surgery represent a significant burden for hospital surgical services, for example, approximately 30,000 patients undergo emergency laparotomy and 65,000 patients require hip fracture surgery in the UK each year with significant morbidity and mortality (Peacock et al. 2018; Perry et al. 2016).

Anaemia is likely to be common in patients undergoing non-elective surgery, as these patients are elderly with multiple comorbidities (Peden 2011; Partridge et al. 2013). Given it is not possible to easily address anaemia preoperatively in these patients, management of anaemia after emergency surgery may translate into improved functional recovery and health-related quality of life (HRQoL). Observational data from the hip fracture population suggests that anaemia impedes functional recovery and increases length of stay (LOS) and re-admission rates (Halm et al. 2004; Foss et al. 2008).

There is significant interest in understanding the optimal use of intravenous (IV) iron in patients undergoing elective surgery (Richards et al. 2015; Munoz et al. 2017), but again less attention has been devoted to the non-elective/emergency setting.

However, any benefits of IV iron on longer-term recovery and HRQoL have to be balanced against potential risks such as infection There remains an ongoing debate about the relationship between iron and infection risk as IV iron administration can increase levels of circulating free iron which can exacerbate pathogen growth and lead to organ dysfunction (Suffredini et al. 2017; Parkkinen et al. 2000). However, importantly, these issues of infection risk will be emphasised in patients undergoing emergency surgery where the background rate of infection can be as high as 40% (GlobalSurg 2018).

We therefore conducted a systematic review to investigate the safety and efficacy of IV iron specifically in patients undergoing non-elective surgery. A better understanding of the existing evidence will help inform clinical practice and/or the design of future clinical trials.

## Methods

This systematic review was performed according to a pre-defined protocol registered on PROSPERO (CRD42018096288), and we followed the Preferred Reporting Items for Systematic Reviews and Meta-Analyses (PRISMA) (Moher et al. 2009). Inclusion criteria were as follows:

(i) Randomised controlled trials (RCT)
(ii) Patients undergoing *non-elective* surgery, defined by the National Confidential Enquiry into Patient Outcome and Death (NCEPOD) as a decision to operate within days (expedited), hours (urgent) or minutes (immediate)
(iii) IV iron, given at any time in the perioperative period (i.e. pre-/intra-/postoperatively), versus comparator

Our search strategy is available in Additional file 1. Two review authors independently screened citations from the systematic search, extracted data, and assessed risk of bias using the Cochrane Collaboration tool (Higgins et al. 2011).

Predefined primary outcomes were as follows:

(i) All-cause infection
(ii) Mean difference in haemoglobin (Hb) concentrations between treatment groups: ‘short-term’ (≤ 7 days), ‘medium-term’ (8–21 days) and ‘long-term’ (> 21 days)

Secondary outcomes were as follows:

(i) Transfusion requirements during study period
(ii) Proportion of participants diagnosed with iron deficiency perioperatively
(iii) Hospital LOS
(iv) Changes in HRQoL
(v) Mortality: ‘short-term’ (≤ 30 days) and ‘long-term’ (> 30 days)
(vi) In-hospital adverse events: anaphylaxis, medical and surgical complications as defined by study authors (e.g. stroke, myocardial infarction, pulmonary embolus, reoperation).

Meta-analysis was performed using a random effects model where enough data were available. For continuous measures, we used the mean difference (MD) with 95% confidence intervals (CI) between treatment arms at follow-up. Dichotomous outcomes were reported as relative risks (RR) with 95% CIs. Heterogeneity was assessed using the *I*^2^ statistic (Higgins et al. 2003). Where haematocrit was reported, values were approximated to haemoglobin using a threefold conversion (Carneiro et al. 2007).

We performed a *post hoc* trial sequential analysis (TSA) to calculate the sample size required to obtain the required statistical power to detect an effect of IV iron on RBC transfusion, which was a primary outcome for two of the included RCTs in hip fracture patients (Serrano-Trenas et al. 2011; Bernabeu-Wittel et al. 2016). We took into consideration the event rate in the control group (40%), a plausible/anticipated relative risk reduction of 20% from the intervention and the anticipated heterogeneity variance (*D*^2^) of the meta-analysis (Wetterslev et al. 2017).

Statistical analyses were performed using Review Manager (RevMan, version 5.3) and TSA program version 0.9 beta (www.ctu.dk/tsa).

## Results

**Fig. 1:**
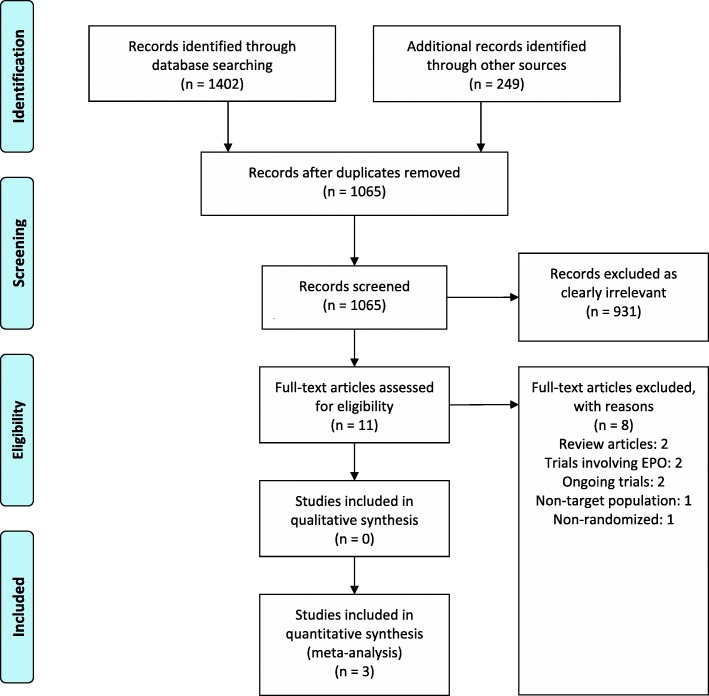
PRISMA diagram

Our study selection process is outlined in the PRISMA diagram (Fig. 1). Of 1065 reviewed studies, three RCTs met our inclusion criteria (Bernabeu-Wittel et al. 2016; Mudge et al. 2012; Serrano-Trenas et al. 2011) and included a total of 605 participants. Details of the included RCTs and interventions are shown in Table 1. Two RCTs administered IV iron preoperatively (Serrano-Trenas et al. 2011; Bernabeu-Wittel et al. 2016) and one administered IV iron postoperatively (Mudge et al. 2012). One trial involved three arms (IV iron + erythropoietin (EPO), IV iron and IV placebo), and we only included data from the IV iron and placebo arms for meta-analysis (Bernabeu-Wittel et al. 2016).

**Table 1:** Characteristics of the included trials

Study	Serrano-Trenas et al. (2011)	Mudge et al. (2012)	Bernabeu-Wittel et al. (2016)
Patient population	Age > 65 undergoing hip fracture surgery	Adults undergoing living-donor or deceased-donor kidney transplantation	Age > 65 requiring hip fracture surgery with Hb levels between 90 and 120 g/L
Country	Spain	Australia	Spain
No. of centres	1	1	13
No. of patients randomised	200	102	303
Intervention	600 mg of IV iron sucrose (Venofer) in three doses of 200 mg at 48-h intervals - First dose always administered before surgery	500 mg of IV iron polymatose on the 4th postoperative day	3 arms • EPOFE—subcutaneous single dose of EPO 40,000 IU and 1000 mg of IV ferric carboxymaltose • FE—1000 mg of IV ferric carboxymaltose and subcutaneous placebo (saline)
Comparator	Usual care	210 mg of oral slow release ferrous sulfate daily until primary endpoint was reached	Subcutaneous single dose placebo and IV placebo (saline)
Primary outcome(s)	• Number and rate of patients transfused postoperatively in each arm	• Resolution of anaemia defined as haemoglobin concentration more than or equal to 11 g/dL for both men and women	• Percentage of patients who received an RBC transfusion during hospitalisation and after 60 days from hospital discharge
Secondary outcome(s)	• Mean number of RBCs per patient • Changes in haematinic variables (admission, 24 h and 7 days postoperatively) • Hospital length of stay • Infection • 30-day mortality • Side-effects	• Gastrointestinal symptoms • Infusion reactions • Acute rejection episodes • Infectious episodes • Blood transfusion • ESA administration	• Number of RBC transfusions per patient • Hb levels 24 and 72 h after surgery, at discharge, and after 60 days • All-cause mortality • Adverse events • HRQoL • Medical complications—acute coronary disease, stroke, heart failure, VTE, COPD exacerbation, deterioration in renal function, infection, delirium, pressure ulcers

*Hb* haemoglobin, *ESA* erythropoiesis-stimulating agents, *EPO* erythropoietin, *HRQoL* health-related quality of life, *RBC* red blood cell

Two trials were carried out in patients undergoing hip fracture (HF) surgery (Serrano-Trenas et al. 2011; Bernabeu-Wittel et al. 2016) and one in patients undergoing kidney transplantation (KT) (Mudge et al. 2012). This trial included a mixture of elective (live donor) and non-elective surgical (cadaveric transplant) patients. We contacted the authors to obtain data relating to cadaveric transplant patients but did not receive a response. Although 70% of participants in this study were undergoing cadaveric transplants, this was not felt to be a clear enough majority by the review team to be included in the meta-analysis. The study was included in risk of bias assessment but its outcome results are presented narratively.

**Fig. 2:**
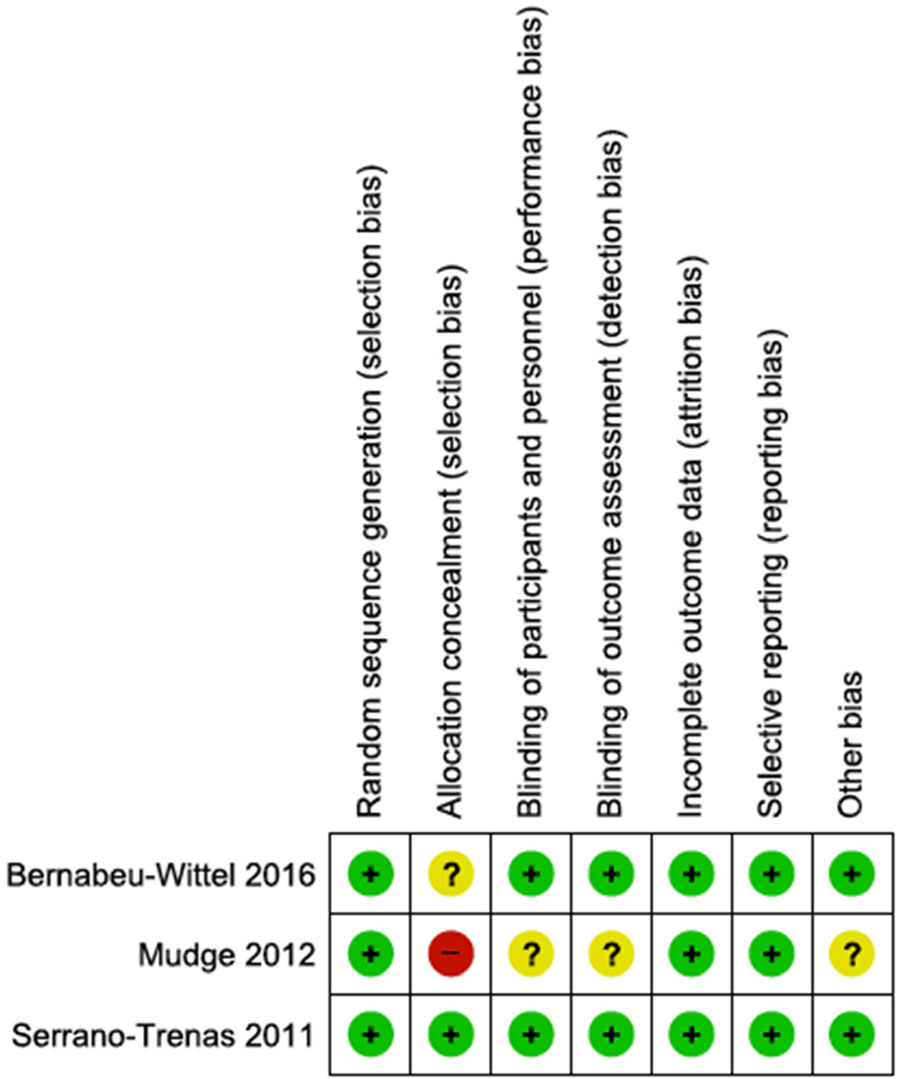
Risk of bias summary for all included trials. **a** Infection. **b** Mean haemoglobin (short-term ≤ 7 days)

The two RCTs in HF participants were generally at low risk of bias across all domains (Fig. 2). Details risk of bias assessments for each trial is provided in Additional file 2.

### Primary outcomes

**Fig. 3:**
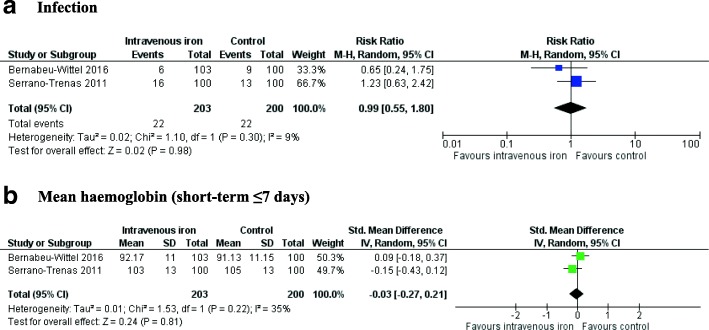
Forest plots of the effect of intravenous iron on primary outcomes. Hb = haemoglobin, CI = confidence interval, M-H = Mantel-Haenszel, IV = inverse-variance. **a** Requirement for RBC transfusion. **b** Mean number of RBCs transfused. **c** Short-term mortality (≤ 30 days)

All three trials reported on infection but none provided a diagnostic definition. Meta-analysis of the two RCTs involving HF patients (Serrano-Trenas et al. 2011; Bernabeu-Wittel et al. 2016) showed no evidence of a difference in the risk of infection in participants who received IV iron compared to those who did not (RR 0.99, 95% CI 0.55 to 1.80, *p* = 0.30, *I*^2^ = 9%) (Fig. 3**)**. The authors of the RCT involving KT patients reported no difference in infection rates in patients who received IV iron compared to oral iron (10 vs. 12, *p* = 0.62) (Mudge et al. 2012).

Meta-analysis of the two RCTs involving HF patients (Serrano-Trenas et al. 2011; Bernabeu-Wittel et al. 2016) showed no evidence of a difference in Hb concentration at ‘short-term’ follow-up (MD − 0.32 g/L, 95% CI − 3.28 to 2.64, *p* = 0.21, *I*^2^ = 37%) (Fig. 3). We were unable to pool the results at our other pre-defined time-points due to variability of reporting in the included trials. One trial observed no difference in mean (± SD) Hb at 60 days post hospital discharge in patients who received IV iron compared to placebo (126.5 (± 15) g/L vs. 119 (± 11.3) g/L, *p* > 0.05) (Bernabeu-Wittel et al. 2016). In RCT involving KT patients, the authors reported no ‘statistically significant difference’ in the median times to resolution of anaemia comparing IV with oral iron (12 days vs. 21 days, hazard ratio 1.22; 95% CI 0.82 to 1.83, *p* = 0.32) (Mudge et al. 2012).

### Secondary outcomes

**Fig. 4:**
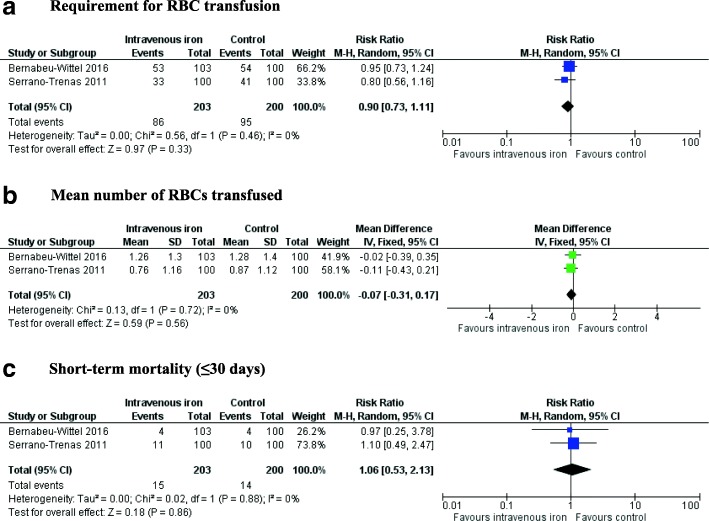
Forest plots of the effect of intravenous iron on secondary outcomes. RBC = red blood cell, Hb = haemoglobin, CI = confidence interval, M-H = Mantel-Haenszel, IV = inverse-variance

**Fig. 5:**
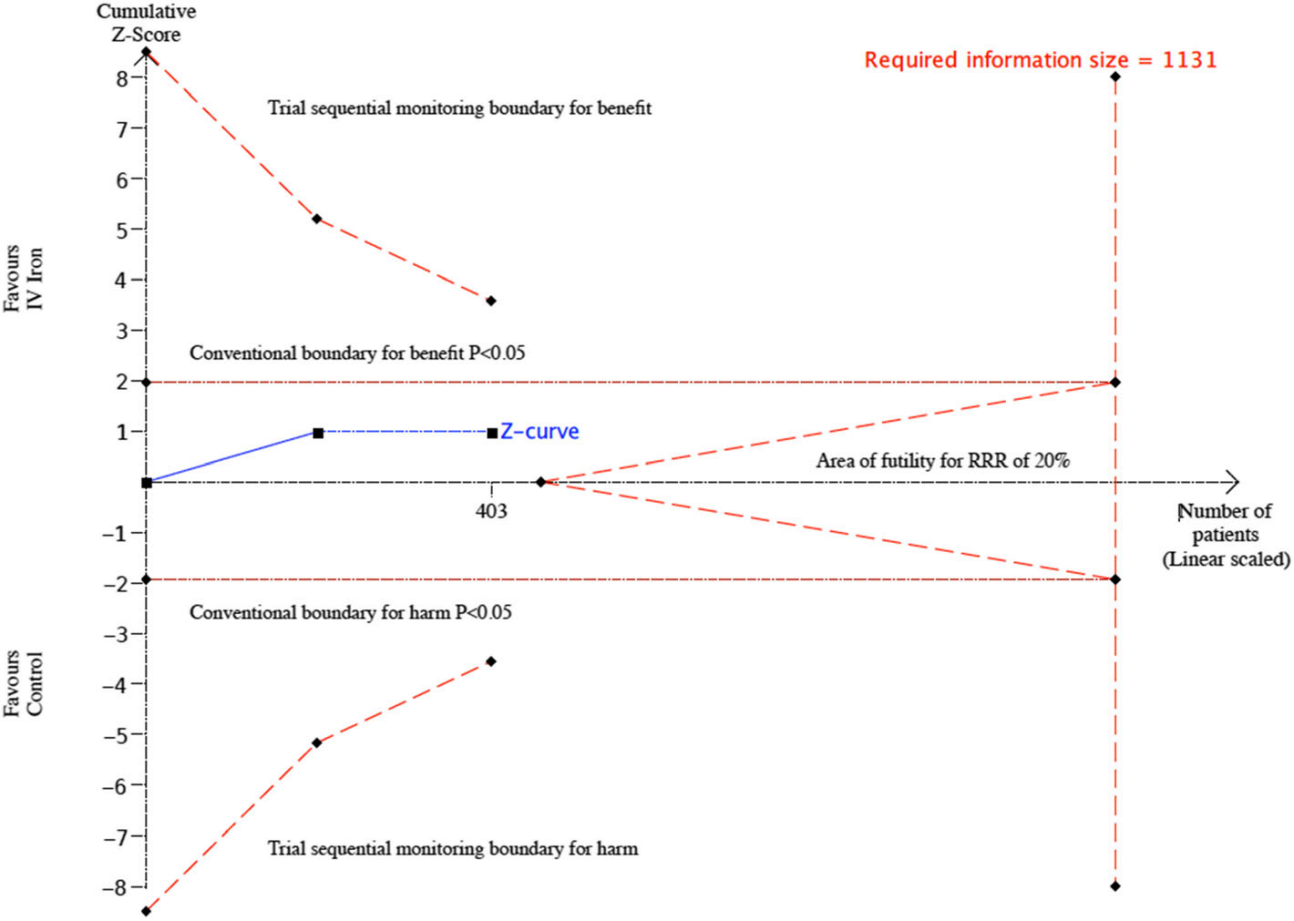
Trial sequential analysis (TSA) of all trials of the effect of IV iron on the risk of requiring a blood transfusion. Control event proportion of 40%, diversity (*D*^2^) of 9%, alpha of 5%, power of 80% and relative risk decrease (RRR) of 20%. The accrued sample size (403) has not reached the required information size (1131)

All three trials reported on the number of participants who required an RBC transfusion. Meta-analysis of two trials involving HF patients (Serrano-Trenas et al. 2011; Bernabeu-Wittel et al. 2016) showed no evidence of an effect of IV iron on the requirement for RBC transfusion (RR 0.90; 95% CI 0.73 to 1.11, *p* = 0.46, *I*^2^ = 0%) (Fig. 4). TSA showed that the required information size to detect or reject an effect of IV iron on requirement for RBC transfusion in patients undergoing HF surgery was 1131 patients, and only 403 were included in this review (Fig. 5). There was also no evidence of a difference in the mean number of RBCs transfused per patients (MD − 0.07, 95% CI − 0.31 to 0.17, *p* = 0.72, *I*^2^ = 0). In the trial involving KT patients, the authors reported no significant difference in the number of patients requiring an RBC transfusion between those who received IV iron compared to oral iron (5 vs. 9, *p* = 0.24) (Mudge et al. 2012).

The two trials in the HF population reported on hospital LOS (Serrano-Trenas et al. 2011; Bernabeu-Wittel et al. 2016). Meta-analysis was not performed as one trial reported mean (± SD) and another median (interquartile range (IQR)). Both trials reported no difference in LOS in participants receiving IV iron compared to placebo − 7 (IQR, 5 to 10) vs. 8 (IQR, 6 to 10) days (*p* > 0.05) (14), 12.9 (± 6.9) vs. 13.5 (± 7.1) days (*p* > 0.05) (16). Only one trial, in patients undergoing HF surgery, measured HRQoL using the Short Form 36 version 2 and found no significant differences among patients receiving IV iron or placebo in physical and mental component scores at 60 days post-discharge (Bernabeu-Wittel et al. 2016). Meta-analysis showed no evidence of an effect of IV iron on ‘short-term’ mortality in patients undergoing HF surgery (RR 1.06; 95% CI 0.53 to 2.13, *p* = 0.88, *I*^2^ = 0%) (Serrano-Trenas et al. 2011; Bernabeu-Wittel et al. 2016) (Fig. 4). One trial provided mortality data at 60 days after hospital discharge, and there no was no difference in the number of HF patients not surviving between the IV iron and placebo groups (12 vs. 10, *p* > 0.05) (Bernabeu-Wittel et al. 2016). All three RCTs reported adverse events (Additional file 3). There were no reported cases of anaphylaxis. Two trials reported gastrointestinal adverse effects with no significant differences between IV iron and placebo or oral iron groups (Serrano-Trenas et al. 2011; Mudge et al. 2012). One trial reported on a range of medical complications between participants receiving IV iron compared to placebo and reported no significant differences (Serrano-Trenas et al. 2011)**.**

We were unable to perform any subgroup analyses due to the lack of available data.

## Discussion

We found no evidence of an effect of IV iron on infection, haemoglobin or transfusion requirements in patients undergoing non-elective surgery. However, the available evidence included only three RCTs that met our inclusion criteria, all with small sample sizes, and the CIs for all outcomes were wide. These limits could encompass clinically important differences. The external generalisability of these RCTs is limited as only two surgical subspecialties were represented (hip fracture, kidney transplantation). Across the included RCTs, there was variability in the dosing and formulations of IV iron used, timing of outcome measurements and availability of data for our pre-specified outcomes.

Our findings are consistent with a recent systematic review assessing the efficacy of postoperative iron in patients undergoing elective surgery (Perelman et al. 2018). The authors identified a larger number of relevant studies but also found no evidence of an effect of iron on transfusion requirements or adverse events (including infection). They did however observe an improvement in Hb in participants who received IV iron but the clinical significance of this was uncertain. Our TSA findings suggest larger RCTs may be warranted to detect an effect of IV iron on transfusion requirements.

Strengths of our review include the strict methodological process, which followed Cochrane Collaboration and PRISMA recommendations. Limitations of our review should be recognised, largely based on the primary trial evidence. Infection was not a pre-defined endpoint in any of the included RCTs, and there were no standardised definitions of infection used. Although we did not observe any differences in mean Hb concentrations between treatment arms, the included RCTs did not specifically target participants diagnosed with iron deficiency.

Interestingly, sustained improvements in Hb up to 60 days after hospital discharge were seen in participants who received EPO and IV iron in one trial (Bernabeu-Wittel et al. 2016), which could be explained by the synergistic effects of exogenous EPO and IV iron. Postoperative inflammation can lead to upregulation of hepcidin—which leads to iron ‘trapping’, blunted erythroid response and decreased EPO production (Ganz 2013; Girelli et al. 2016). Exogenous EPO provides a direct stimulus for erythroid production and reduces hepcidin levels. By reducing hepcidin with EPO and providing supplemental iron, it may be possible to reverse hepcidin-mediated iron dysregulation and thereby provide iron for haemoglobin synthesis (Khorramian et al. 2017). This warrants further investigation especially as inflammation is likely to be present in this cohort of patients.

## Conclusion

In summary, we cannot confirm or refute whether IV iron effects infection, haemoglobin concentration or transfusion requirements in patients undergoing non-elective surgery. Given the high prevalence of anaemia, further well-designed trials across multiple surgical specialties, addressing the limitations we have identified, are required to determine the true safety and efficacy of IV iron ± EPO. Despite observational studies showing an association between anaemia and poor functional recovery, only one RCT measured HRQoL and this indicates the need to include patient-centred outcome measures in future studies. Future trials should include patients identified as being iron-deficient and be powered to address clinically important differences including function and infection, which should be recorded using standardised definitions.

## Additional files

Additional file 1: Search strategy. (DOCX 29 kb)

Additional file 2: Supplemental Digital Content 2: Details of risk of bias assessments. (DOCX 129 kb)

Additional file 3: Supplemental Digitial Content 3: Adverse events reported in the included RCTs. (DOCX 77 kb)

## Abbreviations

CI: Confidence interval
EPO: Erythropoietin
Hb: Haemoglobin
HF: Hip fracture
HRQoL: Health-related quality of life
IV: Intravenous
KT: Kidney transplant
LOS: Length of stay
NCEPOD: National Confidential Enquiry in Patient Outcomes and Death
PBM: Patient blood management
PRISMA: Preferred Reporting Items for Systematic Reviews and Meta-Analyses
RCT: Randomised controlled trial
RR: Relative risk
TSA: Trial sequential analysis
