# Supplementary material for: What is the effect of perioperative intravenous iron therapy in patients undergoing non-elective surgery? A systematic review with meta-analysis and trial sequential analysis
Source: Perioper Med (Lond). 2018 Dec 12;7:30. doi: 10.1186/s13741-018-0109-4 (PMC6290500; doi:10.1186/s13741-018-0109-4)
Supplement: Supplementary file 1 — Search strategy. (DOCX 29 kb) [file 13741_2018_109_MOESM1_ESM.docx]

**PERIOPERATIVE INTRAVENOUS IRON DURING NON-ELECTIVE SURGERY**

**SEARCH NARRATIVE, JUNE 2018**

The following databases were searched for randomised controlled trials and systematic reviews on 5.6.18:

CENTRAL (The Cochrane Library Issue 6, 2018)
MEDLINE (OvidSP, 1946 onwards)

Embase (OvidSP, 1974 onwards)
CINAHL (EBSCOHost, 1982 onwards)

PubMed (epublications ahead of print only)
TRANSFUSION EVIDENCE LIBRARY (1950 onwards)
WEB OF SCIENCE (ISI Conference Proceedings, 1990 onwards)

Ongoing Trial Databases:
ClinicalTrials.gov
WHO International Clinical Trials Registry Platform (ICTRP)

Searches retrieved 1,402 references plus 249 ongoing trials before duplicates were removed, and 845 references plus 220 ongoing trials with duplicates removed, which were sent to the lead author, Akshay Shah, on 7.6.18.

**SEARCH STRATEGIES**

**CENTRAL**
#1 MeSH descriptor: [Ferric Compounds] explode all trees

#2 MeSH descriptor: [Ferrous Compounds] explode all trees

#3 MeSH descriptor: [Iron] explode all trees

#4 (alvofer or colliron or faremio or ferion or feriv or fermed or ferri saccharate or ferric hydroxide sucrose or ferric oxide saccharate or saccharated ferric oxide or ferric saccharate or ferrinemia or ferrisaccharate or ferrivenin or ferrologic or ferrous saccharate or ferrovin or fesin or hemafer s or hemafer-s or idafer or (iron near/2 hydroxide sucrose complex) or iron saccharate or iron sucrose or ironcrose or iviron or nefro-fer or nefrofer or neo ferrum or nephroferol or proferrin or referen or reoxyl or saccharate ferric or saccharate iron or saccharated ferric oxide or saccharated iron oxide or sucro fer or sucrofer or sucroven or veniron or venofer or venotrix)

#5 (anaemex or cosmofer or dexferrum or dexiron or dextrafer or dextran fe or dextran ferrous or dextran iron or driken or fenate or fer dextran or ferric dextran or ferridex or tranferrisat or ferrodex or ferrodextran or ferrous dextran or ferrum lek or fervetag or hibiron or imferdex or imferon or impheron or imposil or infed or infufer or iron dextran or ironate or monofar or proferdex or uniferon or uniferon or uniferron)

#6 #1 or #2 or #3 or #4 or #5

#7 MeSH descriptor: [Administration, Intravenous] explode all trees

#8 (intravenous* or IV or "I.V." or infus* or inject* or parenteral*)

#9 #7 or #8

#10 #6 and #9

#11 (ferric carboxymaltose or Ferinject or Injectafer or Iroprem or ferlecit or ferlixit or ferric gluconate or ferrigluconate or ferrlecit or gluconate ferric sodium or (iron near/2 gluconate) or iron isomaltoside or intravenous iron sucrose or iron sucrose injection* or sodium ferrigluconate or diafer or ferric derisomaltose or venofer or monofer or monafer or monoferro or monover or ferumoxytol or feraheme or rienso or "IV iron" or "I.V. iron" or "iron therapy" or ((intravenous* or inject* or infus* or parenteral) near/3 iron))

#12 #10 or #11

#13 MeSH descriptor: [Perioperative Care] explode all trees

#14 MeSH descriptor: [Perioperative Period] explode all trees

#15 MeSH descriptor: [Specialties, Surgical] explode all trees

#16 MeSH descriptor: [Surgical Procedures, Operative] explode all trees

#17 (preoperat* or postoperat* or perioperat* or operati* or surg* or presurg* or postsurg* or perisurg*)

#18 #13 or #14 or #15 or #16 or #17

#19 #12 and #18

**MEDLINE (OvidSP)**
1. exp Ferric Compounds/

2. exp Ferrous Compounds/

3. exp Iron/

4. (alvofer or colliron or faremio or ferion or feriv or fermed or ferri saccharate or ferric hydroxide sucrose or ferric oxide saccharate or ferric oxide,saccharated or ferric saccharate or ferrinemia or ferrisaccharate or ferrivenin or ferrologic or ferrous saccharate or ferrovin or fesin or hemafer s or hemafer-s or idafer or (iron adj2 hydroxide sucrose complex) or iron saccharate or iron sucrose or ironcrose or iviron or nefro-fer or nefrofer or neo ferrum or nephroferol or proferrin or referen or reoxyl or saccharate ferric or saccharate iron or saccharated ferric oxide or saccharated iron oxide or sucro fer or sucrofer or sucroven or veniron or venofer or venotrix).tw,kf.

5. (anaemex or cosmofer or dexferrum or dexiron or dextrafer or dextran fe or dextran ferrous or dextran iron or driken or fenate or fer dextran or ferric dextran or ferridex or tranferrisat or ferrodex or ferrodextran or ferrous dextran or ferrum lek or fervetag or hibiron or imferdex or imferon or impheron or imposil or infed or infufer or iron dextran complex or ironate or monofar or proferdex or uniferon or uniferon or uniferron).tw,kf.

6. or/1-5

7. exp Administration, Intravenous/

8. (intravenous* or IV or "I.V." or infus* or inject* or parenteral*).tw,kf.

9. 7 or 8

10. 6 and 9

11. (ferric carboxymaltose or Ferinject or Injectafer or Iroprem).tw,kf.

12. (ferlecit or ferlixit or ferric gluconate or ferrigluconate or ferrlecit or gluconate ferric sodium or (iron adj2 gluconate) or sodium ferrigluconate or intravenous iron sucrose or iron sucrose injection* or venofer).tw,kf.

13. (diafer or ferric derisomaltose or iron isomaltoside or monofer or monafer or monoferro or monover or ferumoxytol or feraheme or rienso).tw,kf.

14. (IV iron or "I.V. iron" or iron therapy or ((intravenous* or inject* or infus* or parenteral) adj3 iron)).tw,kf.

15. or/10-14

16. exp Perioperative Care/

17. exp Perioperative Period/

18. exp Specialties, Surgical/

19. (preoperat* or postoperat* or perioperat* or operat* or surg* or presurg* or postsurg* or perisurg*).mp.
20. 16 or 17 or 18 or 19

21. 15 and 20

22. Meta-Analysis.pt.

23. ((meta analy* or metaanaly*) and (trials or studies)).ab.

24. (meta analy* or metaanaly* or evidence-based).ti.

25. ((systematic* or evidence-based) adj2 (review* or overview*)).tw.

26. (evidence synthes* or cochrane or medline or pubmed or embase or cinahl or cinhal or lilacs or "web of science" or science citation index or scopus or search terms or literature search or electronic search* or comprehensive search* or systematic search* or published articles or search strateg* or reference list* or bibliograph* or handsearch* or hand search* or manual* search*).ab.

27. Cochrane Database of systematic reviews.jn.

28. (additional adj (papers or articles or sources)).ab.

29. ((electronic* or online) adj (sources or resources or databases)).ab.

30. (relevant adj (journals or articles)).ab.

31. or/22-30

32. Review.pt.

33. Randomized Controlled Trials as Topic/

34. selection criteria.ab. or critical appraisal.ti.

35. (data adj (abstraction or extraction or analys*)).ab.

36. Randomized Controlled Trial/

37. or/33-36

38. 32 and 37

39. 31 or 38

40. exp Controlled Clinical Trial/

41. (randomi* or trial).tw,kf.

42. (placebo or randomly or groups).ab.

43. exp Controlled Clinical Trials as Topic/

44. or/40-43

45. 39 or 44

46. (Animals/ or exp Animal Experimentation/ or exp Models, Animal/) not Humans/

47. Editorial.pt.

48. 46 or 47

49. 45 not 48

50. 21 and 49

**EMBASE (OvidSP)**

1. ferric carboxymaltose/ or ferric gluconate/ or iron dextran/ or iron isomaltose/ or iron saccharate/

2. (alvofer or colliron or faremio or ferion or feriv or fermed or ferri saccharate or ferric hydroxide sucrose or ferric oxide saccharate or ferric oxide,saccharated or ferric saccharate or ferrinemia or ferrisaccharate or ferrivenin or ferrologic or ferrous saccharate or ferrovin or fesin or hemafer s or hemafer-s or idafer or (iron adj2 hydroxide sucrose complex) or iron saccharate or iron sucrose or ironcrose or iviron or nefro-fer or nefrofer or neo ferrum or nephroferol or proferrin or referen or reoxyl or saccharate ferric or saccharate iron or saccharated ferric oxide or saccharated iron oxide or sucro fer or sucrofer or sucroven or veniron or venofer or venotrix).tw.

3. (anaemex or cosmofer or dexferrum or dexiron or dextrafer or dextran fe or dextran ferrous or dextran iron or driken or fenate or fer dextran or ferric dextran or ferridex or tranferrisat or ferrodex or ferrodextran or ferrous dextran or ferrum lek or fervetag or hibiron or imferdex or imferon or impheron or imposil or infed or infufer or iron dextran complex or ironate or monofar or proferdex or uniferon or uniferon or uniferron).tw.

4. (Ferric carboxymaltose or Ferinject or Injectafer or Iroprem).tw.

5. (ferlecit or ferlixit or ferric gluconate or ferrigluconate or ferrlecit or gluconate ferric sodium or (iron adj2 gluconate) or sodium ferrigluconate or intravenous iron sucrose or iron sucrose injection* or venofer).tw,kf.

6. (diafer or ferric derisomaltose or iron isomaltoside or monofer or monafer or monoferro or monover or ferumoxytol or feraheme or rienso).tw,kf.

7. ferumoxytol/

8. (ferumoxytol or feraheme or rienso).tw.

9. (IV iron or "I.V. iron" or iron therapy or ((intravenous* or inject* or infus* or parenteral) adj3 iron)).tw.

10. 1 or 2 or 3 or 4 or 5 or 6 or 7 or 8 or 9

11. exp surgery/

12. (preoperat* or postoperat* or perioperat* or operat* or surg* or presurg* or postsurg* or perisurg*).mp.

13. 11 or 12

14. 10 and 13

15. Meta Analysis/

16. Systematic Review/

17. (meta analy* or metaanalys*).tw.

18. ((systematic* or literature) adj2 (review* or overview* or search*)).tw.

19. (cochrane or embase or cinahl or cinhal or lilacs or BIDS or science citation index or psyclit or psychlit or psycinfo or psychinfo or cancerlit).ti,ab.

20. ((electronic* or online) adj (sources or resources or databases)).ab.

21. (additional adj (articles or papers or sources)).ab.

22. (reference lists or bibliograph* or handsearch* or hand search* or manual* search*).ab.

23. (relevant adj (journals or articles)).ab.

24. (search term* or published articles or search strateg*).ab.

25. or/15-24

26. (data extraction or selection criteria or inclusion criteria).ab.

27. review.pt.

28. 25 or (26 and 27)

29. editorial.pt.

30. 28 not 29

31. crossover-procedure/ or double-blind procedure/ or randomized controlled trial/ or single-blind procedure/

32. (random* or factorial* or crossover* or cross over* or cross-over* or placebo* or doubl* blind* or singl* blind* or assign* or allocat* or volunteer*).mp.

33. 31 or 32

34. 30 or 33

35. 14 and 34

**CINAHL (EBSCOHost)**
S1 (MH "Surgery, Operative+")

S2 TX preoperat* or postoperat* or perioperat* or operat* or surg* or presurg* or postsurg* or perisurg*

S3 S1 OR S2

S4 (MH "Ferric Compounds+") or (MH "Ferrous Compounds") or (MH "Iron")

S5 TX (alvofer or colliron or faremio or ferion or feriv or fermed or ferri saccharate or ferric hydroxide sucrose or ferric oxide saccharate or ferric oxide,saccharated or ferric saccharate or ferrinemia or ferrisaccharate or ferrivenin or ferrologic or ferrous saccharate or ferrovin or fesin or hemafer s or hemafer-s or idafer or (iron N2 hydroxide sucrose complex) or iron saccharate or iron sucrose or ironcrose or iviron or nefro-fer or nefrofer or neo ferrum or nephroferol or proferrin or referen or reoxyl or saccharate ferric or saccharate iron or saccharated ferric oxide or saccharated iron oxide or sucro fer or sucrofer or sucroven or veniron or venofer or venotrix)

S6 TX (anaemex or cosmofer or dexferrum or dexiron or dextrafer or dextran fe or dextran ferrous or dextran iron or driken or fenate or fer dextran or ferric dextran or ferridex or tranferrisat or ferrodex or ferrodextran or ferrous dextran or ferrum lek or fervetag or hibiron or imferdex or imferon or impheron or imposil or infed or infufer or iron dextran complex or ironate or monofar or proferdex or uniferon or uniferon or uniferron)

S7 S4 OR S5 OR S6

S8 (MH "Administration, Intravenous+")

S9 TX (intravenous* or IV or "I.V." or infus* or inject* or parenteral)

S10 S8 OR S9

S11 S7 AND S10

S12 TX (ferric carboxymaltose or Ferinject or Injectafer or Iroprem or venofer or ferlecit or ferlixit or ferric gluconate or ferrigluconate or ferrlecit or gluconate ferric sodium or (iron N2 gluconate) or sodium ferrigluconate or diafer or ferric derisomaltose or iron isomaltoside or monofer or monafer or monoferro or monover or ferumoxytol or feraheme or rienso or "IV iron" or "I.V. iron" or "iron therapy" or ((intravenous* or inject* or infus* or parenteral) N3 iron))

S13 S11 OR S12

S14 (MH Clinical Trials+)

S15 PT Clinical Trial

S16 TI ((controlled trial*) or (clinical trial*)) OR AB ((controlled trial*) or (clinical trial*))

S17 TI ((singl* blind*) OR (doubl* blind*) OR (trebl* blind*) OR (tripl* blind*) OR (singl* mask*) OR (doubl* mask*) OR (tripl* mask*)) OR AB ((singl* blind*) OR (doubl* blind*) OR (trebl* blind*) OR (tripl* blind*) OR (singl* mask*) OR (doubl* mask*) OR (tripl* mask*))

S18 TI randomi* OR AB randomi*

S19 MH RANDOM ASSIGNMENT

S20 TI ((phase three) or (phase III) or (phase three)) or AB ((phase three) or (phase III) or (phase three))

S21 ( TI (random* N2 (assign* or allocat*)) ) OR ( AB (random* N2 (assign* or allocat*)) )

S22 MH PLACEBOS

S23 MH META ANALYSIS

S24 MH SYSTEMATIC REVIEW

S25 TI ("meta analys*" OR metaanalys* OR "systematic review" OR "systematic overview" OR "systematic search*") OR AB ("meta analys*" OR metaanalys* OR "systematic review" OR "systematic overview" OR "systematic search*")

S26 TI ("literature review" OR "literature overview" OR "literature search*") OR AB ("literature review" OR "literature overview" OR "literature search*")

S27 TI (cochrane OR embase OR cinahl OR cinhal OR lilacs OR BIDS OR science AND citation AND index OR cancerlit) OR AB (cochrane OR embase OR cinahl OR cinhal OR lilacs OR BIDS OR science AND citation AND index OR cancerlit)

S28 TI placebo* OR AB placebo*

S29 MH QUANTITATIVE STUDIES

S30 S14 or S15 or S16 or S17 or S18 or S19 or S20 or S21 or S22 or S23 or S24 or S25 or S26 or S27 or S28 or S29

S31 S3 AND S13

S32 S30 AND S31

**PubMed (epublications ahead of print only)**

#1 (alvofer[TIAB] OR colliron[TIAB] OR faremio[TIAB] OR ferion[TIAB] OR feriv[TIAB] OR fermed[TIAB] OR "ferri saccharate"[TIAB] OR "ferric hydroxide sucrose"[TIAB] OR "ferric oxide saccharate" [TIAB] OR "ferric saccharate"[TIAB] OR ferrinemia[TIAB] OR ferrisaccharate[TIAB] OR ferrivenin[TIAB] OR ferrologic[TIAB] OR "ferrous saccharate"[TIAB] OR ferrovin[TIAB] OR fesin[TIAB] OR "hemafer-s"[TIAB] OR "hemafer-s"[TIAB] OR idafer[TIAB] OR "iron hydroxide sucrose complex"[TIAB] OR "iron saccharate"[TIAB] OR "iron sucrose"[TIAB] OR ironcrose[TIAB] OR iviron[TIAB] OR "nefro-fer"[TIAB] OR nefrofer[TIAB] OR "neo ferrum"[TIAB] OR nephroferol[TIAB] OR proferrin[TIAB] OR referen[TIAB] OR reoxyl[TIAB] OR "saccharate ferric"[TIAB] OR "saccharate iron"[TIAB] OR "saccharated ferric oxide"[TIAB] OR "saccharated iron oxide"[TIAB] OR "sucro fer"[TIAB] OR sucrofer[TIAB] OR sucroven[TIAB] OR veniron[TIAB] OR venofer[TIAB] OR venotrix[TIAB] OR anaemex[TIAB] OR cosmofer[TIAB] OR dexferrum[TIAB] OR dexiron[TIAB] OR dextrafer[TIAB] OR "dextran fe"[TIAB] OR "dextran ferrous"[TIAB] OR "dextran iron"[TIAB] OR driken[TIAB] OR fenate[TIAB] OR "fer dextran"[TIAB] OR "ferric dextran"[TIAB] OR ferridex[TIAB] OR tranferrisat[TIAB] OR ferrodex[TIAB] OR ferrodextran[TIAB] OR "ferrous dextran"[TIAB] OR "ferrum lek"[TIAB] OR fervetag[TIAB] OR hibiron[TIAB] OR imferdex[TIAB] OR imferon[TIAB] OR impheron[TIAB] OR imposil[TIAB] OR infed[TIAB] OR infufer[TIAB] OR "iron dextran"[TIAB] OR ironate[TIAB] OR monofar[TIAB] OR proferdex[TIAB])

#2 (intravenous*[TIAB] OR IV[TIAB] OR "I.V."[TIAB] OR infus*[TIAB] OR inject*[TIAB] OR parenteral*[TIAB])

#3 #1 AND #2

#4 ("ferric carboxymaltose"[TIAB] OR Ferinject[TIAB] OR Injectafer[TIAB] OR Iroprem[TIAB] OR venofer[TIAB] OR ferlecit[TIAB] OR ferlixit[TIAB] OR "ferric gluconate"[TIAB] OR ferrigluconate[TIAB] OR ferrlecit[TIAB] OR "gluconate ferric sodium"[TIAB] OR " iron gluconate"[TIAB] OR "sodium ferrigluconate"[TIAB] OR diafer[TIAB] OR "ferric derisomaltose"[TIAB] OR "iron isomaltoside"[TIAB] OR monofer[TIAB] OR monafer[TIAB] OR monoferro[TIAB] OR monover[TIAB] OR ferumoxytol[TIAB] OR feraheme[TIAB] OR rienso[TIAB] OR "IV iron"[TIAB] OR "I.V. iron"[TIAB] OR "intravenous iron"[TIAB] OR "iron injection"[TIAB] OR "injectable iron"[TIAB] OR "iron infusion"[TIAB] OR "iron infusions"[TIAB] OR "infused iron"[TIAB] OR "parenteral iron"[TIAB] OR "iron therapy"[TIAB])

#5 #3 OR #4

#6 (preoperat* OR postoperat* OR perioperat* OR pre-operat* OR post-operat* OR peri-operat* OR operate OR operated OR operating OR operation OR operations OR surgery OR surgeries OR surgical* OR presurg* OR postsurg* OR perisurg* OR pre-surg* OR post-surg* OR peri-surg*)

#7 #5 AND #6

#8 (random* OR blind* OR "control group" OR placebo* OR controlled OR groups OR trial* OR "systematic review" OR "meta-analysis" OR metaanalysis OR "literature search" OR medline OR pubmed OR cochrane OR) AND (publisher[sb] OR inprocess[sb] OR pubmednotmedline[sb])

#9 #7 AND #8

**TRANSFUSION EVIDENCE LIBRARY**
Clinical Specialty: Surgery
AND
(iron OR ferrous OR ferritin OR ferric) AND (intravenous OR intravenously OR IV OR I.V. OR infused OR infusion OR inject OR injection OR parenteral)

**WEB OF SCIENCE – Conference Proceedings Citation Index (CPCI-S)**#1 TS=(preoperat* OR postoperat* OR perioperat* OR pre-operat* OR post-operat* OR peri-operat* OR operate OR operated OR operating OR operation OR operations OR surgery OR surgeries OR surgical* OR presurg* OR postsurg* OR perisurg* OR pre-surg* OR post-surg* OR peri-surg*)

#2 TS=(iron OR ferritin OR ferrous OR ferric)

#3 TS=(randomized OR randomised OR randomly OR controlled trial OR controlled study OR control group OR control groups OR double blind OR blind study OR blind trial)
#4 #1 AND #42 AND #3

**ClinicalTrials.gov**
Search Terms: preoperative OR postoperative OR perioperative OR operation OR surgery OR surgical OR presurgery OR postsurgery OR perisurgery
Intervention: iron OR ferritin OR ferrous OR ferric
Type of Studies: Interventional

**WHO ICTRP**
Title: preoperative OR postoperative OR perioperative OR operation OR surgery OR surgical OR presurgery OR postsurgery OR perisurgery
Condition:
Intervention: iron OR ferritin OR ferrous OR ferric
Recruitment: ALL
