# Supplementary material for: What is the effect of perioperative intravenous iron therapy in patients undergoing non-elective surgery? A systematic review with meta-analysis and trial sequential analysis
Source: Perioper Med (Lond). 2018 Dec 12;7:30. doi: 10.1186/s13741-018-0109-4 (PMC6290500; doi:10.1186/s13741-018-0109-4)
Supplement: Supplementary file 2 — Supplemental Digital Content 2: Details of risk of bias assessments. (DOCX 129 kb) [file 13741_2018_109_MOESM2_ESM.docx]

**Supplemental Digital Content 2**

*Bernabeu-Wittel 2016*

| Bias | Authors' judgement | Support for judgement |
| --- | --- | --- |
| Random sequence generation  (selection bias) | Low risk | Randomisation stratified by centre and performed by unequal blocks technique. |
| Allocation concealment  (selection bias) | Unclear risk | Details not provided |
| Blinding of participants and personnel (performance bias) | Low risk | Opaque infusion sleeves used to blind clinicians and participants. |
| Blinding of outcome assessment  (detection bias) | Low risk | Not reported but primary outcome is objective. |
| Incomplete outcome data (attrition bias) | Low risk | Reasons for missing data reported and balanced across both groups. |
| Selective reporting (reporting bias) | Low risk | Outcomes reported in manuscript match those specified in pre-published protocol. |
| Other bias | Low risk | No financial conflict of interest identified. |

*Mudge 2012*

| Bias | Authors' judgement | Support for judgement |
| --- | --- | --- |
| Random sequence generation  (selection bias) | Low risk | Computer generated sequence with blocks of 10 to ensure equal allocation. |
| Allocation concealment  (selection bias) | High risk | Sequentially numbered opaque sealed envelopes. However, envelopes were opened by a trial investigator. |
| Blinding of participants and personnel (performance bias) | Unclear risk | Open label trial. IV infusion is challenging to blind. However primary outcome measure is objective and unlikely to be influenced by blinding. |
| Blinding of outcome assessment  (detection bias) | Unclear risk | No details provided |
| Incomplete outcome data (attrition bias) | Low risk | Reasons for missing data reported and balanced across both groups. |
| Selective reporting (reporting bias) | Low risk | Outcomes reported in manuscript match those specified in pre-published protocol. |
| Other bias | Unclear risk | No other sources of bias identified. |

*Serrano-Trenas 2011*

| Bias | Authors' judgement | Support for judgement |
| --- | --- | --- |
| Random sequence generation  (selection bias) | Low risk | Block randomisation in blocks of 10. |
| Allocation concealment  (selection bias) | Low risk | Sequentially numbered opaque sealed envelopes. |
| Blinding of participants and personnel (performance bias) | Low risk | Unblinded study however authors state study drug is challenging to blind. Furthermore, outcome measures are objective and unlikely to be influeced by blinding. |
| Blinding of outcome assessment  (detection bias) | Low risk | Independent evaluator used to perform outcome assessment. |
| Incomplete outcome data (attrition bias) | Low risk | Reasons for participant withdrawal reported in both groups and appear to be balanced. |
| Selective reporting (reporting bias) | Low risk | No pre-published trial protocol. Outcomes listed in 'Methods' are all reported. Two subgroup analyses (intracapsular fractures, those with Hb >12 g/dL) were not prespecified but the authors in the Discussion acknowledge this. |
| Other bias | Low risk | No financial conflict of interest identified. |
