# Supplementary material for: What is the effect of perioperative intravenous iron therapy in patients undergoing non-elective surgery? A systematic review with meta-analysis and trial sequential analysis
Source: Perioper Med (Lond). 2018 Dec 12;7:30. doi: 10.1186/s13741-018-0109-4 (PMC6290500; doi:10.1186/s13741-018-0109-4)
Supplement: Supplementary file 3 — Supplemental Digitial Content 3: Adverse events reported in the included RCTs. (DOCX 77 kb) [file 13741_2018_109_MOESM3_ESM.docx]

**Supplemental Digital Content 3: Adverse events reported in the included RCTs**

| Study | Intervention | Comparator |
| --- | --- | --- |
| Bernabeu-Wittel 2016 | Intervention: IV iron  Adverse events (no. of patients)  Nausea 3; Constipation 3; Hypotension 2; Diarrhea 1, Low grade fever 2; Cholestasis 1; Epigastralgia 1; Peripheral phlebitis 0  Medical complications (no. of patients)  Acute coronary disease 1, Stroke 1, Heart failure 9, VTE 5, COPD exacerbation 6, Renal function deterioration 30, Delirium 25, Skin pressure ulcer 5 | Comparator: IV Placebo  Adverse events (no. of patients)  Nausea 3; Constipation 3; Hypotension 1; Diarrhea 1, Low grade fever 0; Cholestasis 1; Epigastralgia 1; Peripheral phlebitis 0  Medical complications (no. of patients)  Acute coronary disease 2, Stroke 1, Heart failure 12, VTE 3, COPD exacerbation 7, Renal function deterioration 25, Delirium 26, Skin pressure ulcer 8 |
| Mudge 2012 | Intervention: IV iron  Gastrointestinal adverse effects defined as nausea, vomiting, abdominal cramping, constipation, diarrhea  N=3 | Comparator: Oral iron  Gastrointestinal adverse effects defined as nausea, vomiting, abdominal cramping, constipation, diarrhea  N=6 |
| Serrano-Trenas 2011 | Intervention: IV iron  Drug-related side effects  Skin rash 1, General discomfort 2 | Comparator: Usual care  Non reported |
